# Transforming Perceptions: The Development of Pre-pack Regulations in England and Wales

**DOI:** 10.1093/ojls/gqac026

**Published:** 2022-10-29

**Authors:** Bolanle Adebola

**Affiliations:** Associate Professor of Law, University of Reading

**Keywords:** Pre-pack, Corporate Rescue, Pre-pack Regulations 2021, Evaluator, Administration Regulations 2021, Pre-pack Reform

## Abstract

The article systematically assesses the extent to which the Administration (Restrictions on Disposal etc. to Connected Persons) Regulations 2021 achieve the goal of the government to quell the negative perceptions of pre-pack administration. The pre-pack has generated much criticism from disenfranchised groups who regard the practice with much suspicion. These criticisms have triggered questions as to whether and how to structure the regulation of pre-packs.

The article introduces original frames through which to distinguish the competing regulatory visions of the pre-pack, as well as to systematically evaluate the regulatory frameworks that have been introduced. The evaluation reveals a gap between the regulatory visions of the critics and the regulator. This gap has impacted the reception and effectiveness of successive regulatory frameworks. Combining its frames with the expectation gap theory, the article offers a critical assessment of the 2021 reforms, which address most but not all the criticisms of the pre-pack.

## 1. Introduction

This article systematically assesses the extent to which the new pre-pack regulations in England and Wales can achieve the goal of the government to quell negative perceptions of pre-packs.^1^ It sets out the core concerns that feed these negative perceptions and juxtaposes these to iterative regulatory reform cycles. A pre-pack, short for pre-packaged administration, refers to the practice whereby the sale of a financially distressed entity is agreed before the commencement of the administration procedure through which it is executed.^2^ Though considered a feasible mechanism by which distressed but potentially viable businesses could be salvaged, the pre-pack has generated much criticism from disenfranchised groups, who regard the practice with much suspicion.^3^ These criticisms have triggered questions as to whether and how to structure the regulation of pre-packs.

Introducing original frames by which to interpret competing regulatory visions for the pre-pack held by various stakeholder groups, the article reveals the gaps between these visions. It also provides the lenses by which to deconstruct the acceptability of regulatory reforms introduced in the years leading to the 2021 reforms. The article combines its proposed frames with the expectation gap theory to enable a systematic evaluation of the likelihood that regulatory reforms introduced in 2021 would achieve the government’s vision of transforming negative perceptions of the pre-pack.^4^ It also examines the extent to which the new reforms address the concerns at the core of the negative perceptions of the pre-pack, and reveals the critical concerns left unattended. Finally, it highlights further capacity-building responsibilities that must be developed by the regulator.

Divided into 6 sections, the article commences by providing an overview of the framework of the administration and pre-pack procedures in Section 2. Thereafter, Section 3 unpacks the perceptions of pre-packs held by various stakeholder groups. These perceptions form the foundations of the frames introduced by the article in Section 4 and influence the regulatory visions proposed by various stakeholder groups. Section 4 then examines the pre-pack regulatory system through the frames that have been introduced. Section 5 introduces the expectation gap theory, which provides additional insights that combine with the frames introduced in the previous section to create the lenses through which the 2021 reforms are assessed. The conclusions are set out in Section 6.

## 2. Administration and Its Pre-pack Variant: Legal Framework

The administration procedure was designed, in principle, to maximise value in a financially distressed company for the benefit of its body of creditors.^5^ It is a management-displacing procedure that involves the appointment of an insolvency practitioner, called the administrator, to take over the affairs of a distressed company. The appointment may be made by an application to the court or out of court by filing the appropriate documents.^6^ The administrator is appointed to achieve one of three hierarchical statutory objectives that may result in the rescue of the company, its business or the realisation of assets for the benefit of one or more creditors.^7^

To promote collectiveness, the procedure requires the administrator to send their proposals detailing how the stated statutory objective would be achieved to creditors within eight weeks of their appointment.^8^ The ultimate goal is to obtain a decision on the proposed plan from the creditors and, by so doing, engender inclusion.^9^ However, the administrator may elect not to send a statement of proposals, and in effect not consult the body of creditors, where each creditor is to be paid in full, where no distributions would be made to unsecured creditors other than the prescribed part or where neither the company nor its business can be rescued.^10^

While rules regulating the administration procedure can be found in the Insolvency Act 1986, the same is not true of its pre-pack variant, which was developed by practitioners in the shadow of the law. It refers to a practice whereby the sale of all or part of a distressed company’s business or assets is agreed with a purchaser before administration commences, with the administrator executing the sale through one or more transactions upon or shortly after appointment.^11^ The effect is that the collectiveness and inclusion that characterise the administration procedure are foregone under its pre-pack variant.^12^

Though the pre-pack is a creature of practice, a veneer of oversight may be provided by the court where invited, such as when the administration commences through a court application.^13^ The role of the court to which the application is made is not to review the adequacy of the proposed deal but to ensure that the details of, and reasons for, the pre-pack decision have been clearly outlined in the documents presented.^14^ The court has demonstrated, nevertheless, that it is alive to the fact that the incentive to pre-pack may not be reflected in the reasons adduced in the documents.^15^ It has been critical of tactics designed to limit its ability to carefully consider the reasons for administration that have been presented by the applicants.^16^ The court is involved only where invited and the invitation has been extended in only a limited number of cases.^17^ In the period following the reform of the administration procedure in 2002/3, the use of pre-pack has grown considerably, and with it enduring criticisms that are unpacked in the next section.^18^

## 3. Exploring Pre-pack Perceptions

Successive investigations into the pre-pack have concluded that it appears to be a vital restructuring tool that is used only where necessary.^19^ This perception of the pre-pack contends that speed and confidentiality are at the very heart of the pre-pack, and to undermine these would undermine its efficacy as an instrument of rescue.^20^ An alternative view holds the same speed and confidentiality to be the basis of negative perceptions of the pre-pack.^21^ The persistence of the negative perspective has prompted successive regulatory interventions, which will be considered in Section 4.^22^

To understand the events leading to the regulatory reforms, several reports into the pre-pack must be explored. These include: (i) the Sandra Frisby Report, which was published in 2007/8.^23^ It was commissioned by the Association of Business Recovery Professionals (R3) to determine whether the criticisms of pre-packs at the time were justified;^24^ (ii) Teresa Graham’s 2015 review (the Graham Review), which was commissioned by the coalition government in 2013 to investigate enduring criticisms of the pre-pack;^25^ and (iii) the Insolvency Service’s Pre-pack Report 2020,^26^ which was commissioned by the Insolvency Service to review the impact of the recommendations of the Graham Review pursuant to powers reserved through a sunset clause in the Small Business, Enterprise and Employment Act 2015 (the SBEE Act).^27^ The reports unearth the key concerns at the heart of pre-pack criticisms. These concerns are central to understanding the acceptability of the regulatory responses that were introduced after each round of investigations and reports.

The Pre-pack Report 2020 highlighted two key concerns at the heart of the pre-pack practice: (i) concerns with trust in, and the transparency of, the pre-pack procedure—the transparency concern;^28^ and (ii) concerns around the sale of distressed businesses to connected persons—the connected persons concern.^29^ These two key concerns have largely been raised by creditors with no proprietary rights in the assets of the company—unsecured creditors.^30^

The transparency concern is rooted in the exclusion of unsecured creditors from the pre-pack deal. Until 2021, unsecured creditors had suffered a democratic deficit as they were traditionally excluded from knowledge of, and participation in, pre-pack negotiations.^31^ This, they believed, resulted in the sale having a depressed value, which affected the pay out to creditors. Some support for their arguments can be found in the first empirical investigation into pre-packs led by Sandra Frisby, which found that unsecured creditors received zero pence in the pound in 69% of pre-packs, compared with 63% of traditional administration sales.^32^ Similarly, in 2014, an empirical study into pre-packs that was commissioned by Teresa Graham (the Wolverhampton Report) showed that the average returns to unsecured creditors as a percentage of the debt owed was 7.22% in pre-packs, compared with 13.06% in traditional administrations.^33^

While pre-packs are criticised by unsecured creditors, it is pre-pack sales to connected persons that attract the greatest umbrage. Again, there is justification for their concerns because unsecured creditors fare the worst in pre-pack sales to connected persons. The Wolverhampton Report showed that average returns to unsecured creditors fell to 6.07% in sales to connected persons,^34^ while it improved to 8.82%^35^ where the business was sold to unconnected persons. Both figures still fall short of the returns of 13.06% in traditional administrations, in which unsecured creditors have more influence.^36^ The unsecured creditors’ concerns are exacerbated by the fact that pre-pack sales to connected persons are much more likely to fail within the next three years than sales to unconnected persons. The Graham Review noted that almost 30% of connected party sales failed within 36 months, while about 18% of unconnected sales failed in the same period.^37^

Given the foregoing, it should come as no surprise that the pre-pack elicits concerns about its transparency and connected party sales.^38^ Nevertheless, though the initial empirical investigation into the pre-pack by Frisby observed both concerns, only the transparency concern attracted any attention and a response.^39^ It was not until 2014 that the Graham Review took a more critical approach to both the transparency and connected sales concerns.^40^ To determine whether both concerns had been eliminated in the period following the Graham reforms, the Insolvency Service undertook investigations into the impact of Graham’s recommendations, which it set out in the Pre-pack Report 2020.

At the very heart of the concerns are issues relating to the value obtained for the distressed entity. The Graham Review found that administrators mainly carried out desk-top valuations.^41^ Where the sale was made to connected parties, the valuation figure simply matched the price paid for the business.^42^ Independent valuations were introduced in 2013, which the Graham Review enhanced by recommending that administrators should use the services of valuers who hold professional indemnity insurance or explain their reasons for not doing so.^43^ The Pre-pack Report 2020 found that over 90% of pre-pack sales to connected parties in 2016 obtained independent valuation, with three-quarters utilising valuers with indemnity insurance.^44^ Nevertheless, only a little over half of connected purchasers paid over the valuation price.^45^ In just under half of all purchases, the purchase price matched or fell below the valuation figure; of these, 7% exactly matched the valuation figure and almost 40% sold for less than the valuation figure.^46^ Of the sales made below the valuation figure, a quarter were sold for at least 25% less than the market value.^47^

The low purchase prices pre-packs attracted were attributed, at least in part, to the lack of, or limited marketing of, pre-packs, particularly when sold to connected persons.^48^ The Pre-pack Report 2020 noted that there had been much improvement in the marketing of pre-pack sales, from less than half to slightly over three-quarters of sales.^49^ Still, there was no marketing done in about one in five pre-packs.^50^ Moreover, amongst the slightly more than three-quarters that complied with the marketing principles introduced by the Graham Review in 2015, administrators adhered to only three of the six principles of good marketing that had been introduced.^51^ These three principles related to transparency matters and required the administrator to set out the reasons for their actions.^52^ There was considerably less compliance with the principles actually relating to marketing.^53^ Still, it appeared that the purchase price was not unduly impacted by the presence or absence of marketing. Businesses were sold below the valuation figure in 43% of the cases in which there was no marketing and in 46% of those in which there was marketing.^54^ Creditors nonetheless noted that the knowledge that marketing had been carried out gave them greater confidence that a fair price had been obtained.^55^

As stated above, the Graham Review showed that pre-pack sales were more likely to fail than traditional administrations.^56^ To address this high rate of recidivism, the Graham Review recommended the introduction of an independent viability review,^57^ as well as the establishment of a pool of pre-pack experts (the Pre-pack Pool)^58^ where the sale was to connected persons.

An independent viability review may be obtained from an independent person where the pre-pack sale is to a connected person.^59^ This voluntary statement would show how the company purchasing the distressed business expected to survive over the ensuing 12 months.^60^ The Pre-pack Report 2020 found that only a little over one-quarter of pre-pack sales to previously connected persons clearly included a viability report.^61^ Nevertheless, it did not seem that the lack of a viability statement resulted in the failure of the business post-sale. While 69% of the companies that provided a viability report failed subsequently, 87% of those that did not were still trading after 12 months.^62^ It is important to note, however, that the government’s investigation spanned only a 12-month period. The Wolverhampton Report revealed that the rescued businesses remained vulnerable to failure within 36 months of rescue.^63^ While just over 5% had failed within 12 months, 25.5% had failed by 36 months.^64^ It is thus difficult to draw any conclusions from the Pre-pack Report 2020, which merely examined the first 12 months. The government nevertheless concluded that the inclusion of the viability report should remain a matter of good practice.^65^

It was because the sale to connected persons was perceived to be one of the main characteristics associated with the possible failure of the pre-pack in the future that the Graham Review recommended the establishment of the Pre-pack Pool.^66^ The Pre-pack Pool comprised independent persons with relevant experience, who could be approached voluntarily by connected persons before a sale to give an opinion about the reasonableness of the decision to pre-pack.^67^ At the time of the Graham Review, almost two-thirds of pre-packs sales were to connected persons.^68^ The number dropped to less than half of pre-packs in the first year following the reforms, but this inched back up again in the ensuing years.^69^ Despite connected-party pre-packs accounting for more than one in every two pre-pack sales since 2017, the number referred to the Pre-pack Pool steadily reduced, with less than 10% of such sales being referred from 2018.^70^ The low referral rate was attributed to factors such as the lack of benefit and the cost of the procedure.^71^ Though some stakeholders seemed unaffected by the role of the Pre-pack Pool, others noted that the reviewer’s opinion affected their perceptions of the sale and new company.^72^ Where the Pre-pack Pool had been approached, it provided unsecured creditors with comfort about the reasonableness of the transaction.^73^ Such was the level of comfort provided that some stakeholders requested that the remit of the Pre-pack Pool be extended to other rescue procedures.^74^ The low uptake was thus very concerning.^75^

The Graham Review’s recommendations were set within a comply or explain framework through which insolvency practitioners were to make disclosures to creditors. As will be discussed below, the disclosures were introduced through revisions to the extant Statement of Insolvency Practice (SIP) 16.^76^ The revised SIP 16 was expected to improve transparency by providing information about the pre-pack to the stakeholders. The document would be accompanied by other documents, including the viability review and statement of the Pre-pack Pool, where available. The Pre-pack Report 2020 revealed concerns about compliance. Though there had been an improvement in compliance, the report asserts that there was still room for further improvement.^77^

It follows from the foregoing that the negative perceptions of the pre-pack have not been unfounded. They stem from key concerns to which affected stakeholders expected a response from the regulator. However, while the transparency concern was universally recognised in 2007, the connected persons concern, though present at the same time, was not acknowledged. Thus, while the transparency concern received a prompt regulatory response, its connected persons counterpart received no response until 2015. The Pre-pack Report 2020 shows that these concerns have remained even after the implementation of the Graham Review’s reforms, necessitating further intervention from the government. The next section shows the influence of these concerns on the preferred approach to pre-pack regulation for affected stakeholders.

## 4. Transforming Pre-pack Perceptions: The Development of Pre-pack Regulation

This section explores competing visions of pre-pack regulation and outlines the trajectory of the pre-pack’s regulatory journey. Thereafter, it examines each regulatory reform in light of the outlined competing regulatory visions.

### A. Self-Regulation: Development of Pre-pack Regulatory Visions

Proponents^78^ of the pre-pack concede that the process is undeniably opaque and accept that there should be greater transparency around the deal.^79^ Nonetheless, before 2015, any opportunity for creditors to review the deal was unacceptable to them.^80^ Instead, they argued that unsecured creditors should be informed about the deal after it had been concluded but that the process should not include the power to unravel the settled deal.^81^ Thus, they recommended procedures providing creditors with the right to review the deal after it had concluded. Suggested procedures included exiting the administration procedure via a compulsory liquidation^82^ and/or improving the transparency of the deal through targeted communications to unsecured creditors.^83^

Leading proponents have been associations representing practitioners, including R3 and the Institute of Chartered Accountants in England and Wales (ICAEW).^84^ While their position changed as the government threatened to ban connected-person sales,^85^ which would have affected pre-packs considerably, their historical view remains vital to an understanding of the development of the regulatory framework.

They argued historically that any procedure that breached the confidentiality and speed of the pre-pack process was inimical to its success.^86^ Further, such procedures would lead to a significant drop in the level of pre-packs, to the detriment of secured creditors, employees, entrepreneurs and even the economy.^87^ They argued that the problem of the pre-pack stemmed mainly from the negative perceptions generated by its opacity, negative media coverage and general misunderstanding of the context of corporate insolvency.^88^ Therefore, for them, any regulation ought to focus on the creation of avenues for greater transparency to facilitate better understanding of the reasons for the pre-pack and the deal that was struck. They argued that such information should be provided after completion. This vision is described in this article as an argument for *review rights*.^89^

In contrast, pre-pack critics focused on a different set of fundamental questions. For them, there were two main questions that a regulatory framework should address. The first was whether the decision to pre-pack was rightly made and the second was whether the best value was obtained. Both questions can only be answered on a case-by-case basis before the completion of the deal. Thus, they argued that the appropriate regulatory response should be either to include them in the negotiations or to provide independent oversight of the deal before it is passed. This would enable the scrutiny of each case to determine whether the pre-pack decision was rightly made and whether the best possible deal in the circumstances was achieved. Given their emphasis on pre-sale scrutiny, this vision of pre-pack regulation is described as the argument for *preview rights*.^90^

Some critics within the preview rights vision argue further that preview rights should also carry the power to stop an unfavourable deal, where necessary. An example would be in situations where the best value has not been offered for the assets or where the future viability of the distressed entity is questionable.^91^ Thus, the vision of this subset of critics can be described as *enhanced preview rights* because they demand preview rights enhanced with the right to stop deals, where necessary.^92^ Suggested procedures with the enhanced right of preview include procedures giving the unsecured creditors a pre-approval notice period,^93^ giving the court the responsibility of independent oversight^94^ and appointing a different practitioner to execute the pre-pack from the one that negotiated its terms.^95^

As could be expected, critics primarily consisted of unsecured creditors, who strongly favour enhanced preview rights.^96^ At the forefront of the unsecured creditor position have been three leading unsecured creditor groups: the Association of British Insurers (ABI), the British Property Federation and BPIF.^97^ In 2008, the ABI, which insures suppliers and trade creditors, stated its interest in preview rights when making its case to the Business and Enterprise Committee of the House of Commons.^98^ Further, the ABI, in its response to the 2011 consultations, argued that the unsecured creditors ought to ‘test the assumption’ that the pre-pack and its proceeds were the best deal that could have been obtained in the circumstances.^99^ The Business and Enterprise Committee appeared to agree with the position of the unsecured creditors when it stated that:

The interests of unsecured trade creditors must take a higher priority, especially in ‘phoenix’ pre-pack administrations … Where there are good reasons for an insolvency practitioner agreeing to a pre-pack, which there can often be, this must be explained clearly and fully. Where there are no good reasons for entering a pre-pack, *this must be exposed before the damage is done*.^100^

### B. Pre-pack Regulation: From Review to Preview Rights

The first regulatory response was through an instrument called the Statement of Insolvency Practice (SIP); in particular, SIP 16, which came into effect on 1 January 2009.^101^ SIP 16 is, at its core, a guidance note through which practitioners are required to disclose information about the process leading to, and details of the deal to unsecured creditors.^102^ Practitioners are to disclose the requisite information as soon as reasonably practicable after the sale. SIP 16 was produced by the Association of Business Recovery Professionals, approved by the Joint Insolvency Committee and adopted by each of the recognised regulatory bodies.^103^ Thus, the initial regulatory approach provided a system of self-regulation designed to address the transparency concern around the pre-pack deal.^104^ To promote compliance, the Insolvency Service maintained oversight of the system, publishing its empirical findings intermittently.^105^ Given the nature of the instrument, a breach does not attract a legal penalty. Instead, disciplinary action could be taken by the relevant regulatory body of which the practitioner was a member.^106^ SIP 16 has been strengthened over the period of its existence through the expansion of the range of information provided and the provision of additional guides to aid compliance with its ethos.^107^ Its effectiveness is also augmented, in principle, by SIP 13, which regulates sales to connected persons and has similarly been revised.^108^

While the self-regulatory regime introduced in 2009 addressed the transparency concerns around the pre-pack, they did not quell the negative perceptions of pre-packs. As has been said above, the persistence of the negative perceptions led to the commissioning of the Graham Review in 2013.^109^ Empirical revelations made in the course of the review supported the claim of unsecured creditors that the process required additional independent oversight.^110^ In response, the Graham Review recommended a menu of reforms, including the independent valuation report,^111^ the independent viability report^112^ and a marketing template,^113^ to direct actors on how to execute the deal.^114^ For the first time, independent oversight was introduced, to be provided by the Pre-pack Pool.^115^ In contrast to the 2009 response following the Frisby Report, the Graham Review addressed both the transparency and connected persons concerns. Nevertheless, it maintained the self-regulatory approach introduced through SIP 16.^116^

The Pre-pack Pool comprises a body of business experts who review pre-pack deals when approached.^117^ The Pre-pack Pool applied only to pre-pack sales to connected persons. Recall that these were considered to produce the worst outcome for unsecured creditors.^118^ The process was voluntary. The connected person provided the information listed on the Pre-pack Pool’s website and paid the stated fees. The case file was randomly allocated to a reviewer, who returned an opinion. The applicant was to inform the administrator of the outcome and provide the Pre-pack Pool’s opinion for onward communication to the stakeholders.^119^

Additional guidance for pre-packs was set out in the Insolvency Code of Ethics.^120^ The Code is essentially a set of principles and best practice standards to which all practitioners must adhere.^121^ Like the SIP, breach of the Code is not actionable. However, it is a factor that may be taken into consideration when decisions are to be made on the actions of the practitioner.^122^ The Code sets out five fundamental principles to which the practitioner must adhere, including objectivity, integrity, professional competence, due care, confidentiality and professional behaviour. The practitioner is proactively to identify actual or potential threats to compliance with these principles that arise in the lead up to or upon appointment. After carefully evaluating these threats, the practitioner should take reasonable steps to mitigate or eliminate them.

Specifically, pre-packs have been identified as threatening the principle of objectivity, particularly by those excluded from the process of sale, such as unsecured creditors.^123^ The Code therefore invites the practitioner to mitigate the threats to the principle of objectivity by following due process through, for example, obtaining an independent valuation of the assets or business being sold, or consulting with other potential purchasers, where possible.^124^ Such steps would also align with expectations under SIP 16. In fact, the practitioner demonstrates adherence to the principle of professional competence and due care by ensuring continuing awareness and understanding of relevant developments with law and practice, including changes to SIP 16.^125^

From the foregoing, we can observe a gradual but reluctant shift in pre-pack regulation over the first decade of its existence. In that time, the insistence on review rights by pre-pack proponents was overtaken by the provision of preview rights favoured by critics. The latter reforms also addressed the connected persons concern, in addition to the transparency concern that was the centre of initial regulatory interventions. At first blush, the Pre-pack Pool seemed an excellent innovation because it provided preview rights without compromising on the speed, confidentiality, and cost-effectiveness of the pre-pack process. Further examination below reveals that the preview rights provided by the Pre-pack Pool operated within strict confines and fell short of the enhanced preview rights advocated by unsecured creditors.^126^

#### (i) Preview rights versus enhanced preview rights: oversight of pre-pack decision

The role of the Pre-pack Pool reviewer was to examine the justifications for the decision to pre-pack, to determine whether they supported the case for a pre-pack.^127^ The restriction of their role can be traced to the position of proponents that practitioners resort to pre-packs only when it is impossible to preserve value in the business through the more inclusive traditional administration. Given that the decision to pre-pack ought to be taken on a case-by-case basis, the Pre-pack Pool was to determine whether the case was made on each pre-pack before it. For each reviewed case, therefore, the Pre-pack Pool reviewer made one of the following three statements: (i) that nothing suggests that the grounds for pre-pack are unreasonable (positive); (ii) that more information is required, but nothing suggests that the ground for the pre-pack are unreasonable (qualified positive); or (iii) that there is lack of evidence to support a statement that the grounds for the pre-pack are reasonable (negative).^128^ Between 2015 and 2020, the Pre-pack Pool made 63 positive, 24, qualified positive and 18 negative statements.^129^

In its 2018 Report, the Pre-pack Pool informed us that 30% of the companies it had reviewed were still trading after 14 months, including one that had received a negative report.^130^ The other five referrals that received a negative report had all failed within the same time.^131^ This begs the reader to question the extent to which the Pre-pack Pool reviewer could successfully execute even the Pre-pack Pool’s narrowly construed role. It is difficult for reviewers to reach behind the carefully constructed explanations presented by previously connected persons to identify unjustifiable pre-packs, hence the need for additional information. Thus, it becomes imperative to understand the additional incentives key stakeholders have to choose the pre-pack as opposed to the more inclusive traditional administration.

Connected persons are more likely to be the main purchasers of the distressed business in the case of pre-packs than in the case of traditional administrations. The Frisby Report found that 59% of the pre-packs in the sample were sold to connected persons.^132^ It also found that pre-packs have been on the rise since the introduction of insolvency law reforms in 2002/3.^133^ Thus, while pre-Enterprise Act 2002 connected sales were 53%, post-Enterprise Act pre-pack sales to connected persons increased to 62%.^134^ Similar to the post-Enterprise Act results, the Wolverhampton Report found that 63.3% pre-packs were connected sales, while over 30% of traditional administration business sales were to connected persons.^135^ The Pre-pack Report 2020 reveals that the volume of pre-packs dropped following the 2015 Graham intervention. Nevertheless, it was still the case that more than half of all pre-pack sales were to connected persons.^136^ It is thus reasonable to infer that when connected persons choose to purchase their businesses, they opt for the pre-pack route, not traditional administration, regardless of the explanation that is given.

It is easy to understand their preference. The pre-pack gives them control of the negotiations and its outcomes, including the price to be paid for the business. They enjoy asymmetric informational and procedural advantages.^137^ The Frisby Report in 2007, as well as the Wolverhampton Report in 2014, showed that pre-packs were poorly marketed.^138^ While the Frisby Report stated that most pre-packs had some form of valuation done, it was not clear how such reports were obtained.^139^ The Wolverhampton Report showed that desktop valuations were typically conducted, and that these matched the sum offered by the buyers to the sums owed to the secured creditors, with a little left over to fund the procedure.^140^ Though practitioner groups argue that speed and secrecy are imperative in the case of the pre-pack, in the absence of which there would be a loss of goodwill, the Wolverhampton Report curiously showed that goodwill was typically left out of the valuation.^141^

Since 2003, directors have been responsible for most administrator appointments.^142^ Given their position, they can shop for administrators who would execute the rescue through the pre-pack. Still, we know that insolvency practitioners are well regulated and professional.^143^ They are required to exercise independent judgment upon appointment.^144^ In any event, connected parties are not repeat players, so it is unlikely that they can ‘capture’ the practitioners.^145^ Further, while the directors appoint the practitioners, they usually do so with the consent or acquiescence of the secured lender.^146^ Thus, one may argue that the fact that connected persons would prefer to pre-pack does not mean that they can attain that outcome because the administrators could prefer a different route. Even practitioners acknowledge some discomfort in relation to pre-pack sales to previously connected persons, though they support the pre-pack procedure more generally.^147^ It is thus imperative that we examine the incentives of the most powerful of the stakeholders: those with proprietary rights in the debtor—ie secured creditors—who ultimately determine the direction of the administration.

It is well known that administrators acquiesce to the wishes of secured creditors.^148^ While it may appear that secured creditors have no reason to prefer a pre-pack to a traditional sale, the Frisby Report showed that they fared better on average in traditional administrations, where they received 68.8% of the sums owed, than they did in pre-packs, where they received 66.7%.^149^ In both procedures they were likely to receive up to 75% of the sums owed at least 64% of the time.^150^ Further examination of the figures, however, reveals a difference that would justify their preference for pre-packs. The Frisby Report showed that secured creditors were more likely to receive 100% of the sums owed through pre-pack proceedings, where about half (49%) of all cases yielded 100%, than they would in the traditional administration, where only about one-third (37%) of the cases yielded 100% dividend.^151^

Thus, with the pre-pack, we are faced with a rescue procedure favoured by the principal actors in the process who, collectively, have absolute control. On the rare occasion that an application was made to the Pre-pack Pool, they would have no difficulty in providing an explanation that fitted their decisions.^152^ On the other hand, there are unsecured creditors who are impacted by the outcomes but excluded from the process. Even worse, they fare worse in pre-packs sales than in traditional administrations.

#### (ii) Preview rights v enhanced preview rights: oversight of pre-pack deal

As stated above, unsecured creditors wanted preview rights that extended to the deal itself, coupled with the power to stop an unacceptable deal; a right that is available, at least in principle, in a traditional administration.^153^ Approving the deal involves accepting the valuation of the business and price offered. It is valuation that determines whether and to what extent unsecured creditors can participate in the outcomes of the business sale. Practitioners note that unsecured creditors tend to question the disparity between the stated value of the assets prior to administration and the outcomes of the pre-pack sale.^154^ They explain that much of the value of the business tends to be tied up in its brand and goodwill, which diminish rapidly following the entry into administration. Oversight of the valuation decision was therefore fundamental to changing pre-pack perceptions.^155^

Recall that poor valuation practice in pre-packs was highlighted by the Graham Review’s report as one of the major failings of the process in 2014.^156^ Given the absence of competing bids, in many pre-packs, both SIP 16 and the Ethics Code recommended obtaining an independent valuation of the business, against which the proposals received could be evaluated. While the Pre-pack Pool reviewer would examine the valuation report where presented, there was no obligation for previously connected persons to present one.^157^ Even where a valuation report was presented, the Pre-pack Pool reviewer was unlikely to have a comparator against which to make a judgment because the putative administrator was not required to submit the independent valuation they obtained for the business, or competing bids and valuations, to the surprise of some practitioners.^158^ It clearly would be difficult for the Pre-pack Pool reviewer to understand the disparity between the value offered by the previously connected person and the valuation of other, independent valuers.^159^ Even where the independent valuation was submitted, the Pre-pack Pool reviewer was not required to make a statement on its adequacy. In any event, the pre-pack sale could still go forward even with a negative comment from the Pre-pack Pool.^160^

It follows from the foregoing that the Pre-pack Pool was unable to examine a deal in any detail, let alone halt bad deals.^161^ Thus, there was a gap between the preview rights offered by the Pre-pack Pool and the enhanced preview rights envisaged by the unsecured creditors. This gap posed existential problems for the Pre-pack Pool. In the situation where the Pre-pack Pool issued a positive opinion, the reputational effects of that opinion went beyond the carefully worded limits that had been drawn by the regulators. It sent the message to the market that the *deal*, not merely the *decision* to pre-pack, *was rightly made*.

A perfect example of the challenge the Pre-pack Pool faced can be found in the case of the Polestar Group. Polestar was the UK’s largest publication printer, printing up to 50 million well-known magazines a week, including *Hello*, *Cosmopolitan* and *Grazia*.^162^ Since its establishment in 1998, it had been through several iterations, including two infamous pre-packs within a decade of its final pre-pack in 2016.^163^ Following inadequate financial restructuring in 2015, it sought a pre-pack in March 2016 that would see the business sold to a wholly owned subsidiary of one of its principal shareholders, Proventus Capital partners.^164^ Given that they were previously connected buyers, they approached the Pre-pack Pool for a statement.^165^ Within the limits of its role, the Pre-pack Pool reviewer examined the decision to pre-pack and gave a positive statement.^166^ The pre-pack was completed on the day that the company was put into administration.^167^ Following the pre-pack, its three largest customers refused to novate their businesses to the new company, precipitating its return to administration and ultimate failure.^168^

The case has been described as the *bête noire* of the Pre-pack Pool.^169^ However, the case was neither anomalous nor difficult to understand. The Pre-pack Pool had successfully executed its limited role, which was to consider the decision to pre-pack with which it was presented. It had given an answer that could not adequately take into consideration the broader issues that concerned the other stakeholders who were impacted by the pre-pack deal because its role did not extend that far. The Polestar Group was suffering viability issues as a result of changes in its industry.^170^ Though the Pre-pack Pool was successful in its role—deciding whether the decision to pre-pack had been rightly made (preview rights)—it had been unsuccessful in the role expected of a body with enhanced preview responsibilities, as argued above.^171^ In the absence of enhanced preview rights, it could neither examine the quality of the deal nor stop it from going forward if it considered the deal to be poor.^172^

The voluntary, self-regulatory approach to pre-pack regulation has clearly been unsuccessful. Although the idea of the Pre-pack Pool promised much, it delivered little. Essentially, it was a benign solution to fundamental pre-pack concerns. While governments are encouraged to employ self-regulation technologies to improve compliance, they are also enjoined to recognise that this approach has its weaknesses; particularly, that the regulated are not always willing to comply.^173^ Where self-regulation methods have failed to eliminate negative behaviour, governments are advised to escalate to more command-oriented instruments.^174^ In the case of pre-packs, the Administration (Restrictions on Disposal etc to Connected Persons) Regulations 2021 (Administration Regulations 2021) were introduced.

### C. The Administration (Restrictions on Disposal etc to Connected Persons) Regulations 2021: From Self-Regulation to Mandatory Regulations

The Administration Regulations 2021 came into effect on 30 April 2021.^175^ They prohibit the administrator from making a substantial disposal of the company’s assets to connected persons within eight weeks of commencement, unless they have received either pre-disposal approval from creditors or a qualifying report from an evaluator.^176^ It is for the administrator to determine whether a person is a connected person, or whether a transaction should be designated a substantial disposal.^177^

#### (i) The creditor approval route

The duty to obtain creditor approval lies with the administrator, who must seek a decision using a decision-making procedure. This requires the administrator to send proposals for making the disposal in the statement of administrator’s proposals to creditors.^178^ After considering the proposals, the creditors may approve, reject or recommend modifications acceptable to the administrator.^179^ The administrator is bound by the decision of the creditors.

#### (ii) The qualifying report route

The qualifying report route places responsibility for obtaining a report on connected persons seeking to purchase the business. The qualifying report is obtained from a person known as the evaluator, who need not be an insolvency practitioner.^180^ To meet the requirements as to skill, the evaluator must identify the knowledge and experience upon which they rely to make the report.^181^ The evaluator must also provide information about the professional indemnity insurance taken out by them or on their behalf.^182^ They must be independent and must not belong to any of the prohibited groups.^183^ The qualifying report will be invalid if the evaluator fails to satisfy any requirement as to status.^184^

The qualifying report must meet the requirements as to form and content.^185^ It must also meet the requirements as to substance. The latter requires the evaluator to provide either a case made or a case not made opinion. The case is made where the evaluator states that they are satisfied that the consideration to be provided and the ground for the substantial proposal are reasonable.^186^ The case is not made where the evaluator is not satisfied that the consideration to be provided for the relevant property and the grounds for the substantial disposal are reasonable in the circumstances.^187^ The evaluator must state their principal reasons for making the statement and a summary of the evidence they relied on.^188^

The connected person must present the evaluator’s report to the administrator, who must consider it and satisfy themself that the individual making the report met all formal and substantive requirements and had sufficient knowledge and experience to make such a report.^189^ Unlike the result of the creditors’ decision, the administrator must consider, but is not bound to follow, the evaluator’s report.^190^ They may execute the disposal or accept a competing proposal even where the qualifying report or a previous qualifying report contains a case not made opinion.^191^ The administrator should be guided by the duty to act in the best interests of the creditors.^192^ However, they must explain their decision in the statement sent to creditors.^193^

The Administration Regulations 2021 clearly introduce mandatory oversight of pre-pack decision making. A key consideration is whether escalation from self-regulatory instruments to the mandatory regulations address the pre-pack concerns sufficiently to eliminate the negative perception of the practice. As this section has demonstrated, there is a gap between the regulatory expectations of the critics and those underlying the 2009 and 2015 reforms.^194^ Thus, if the concerns and negative perceptions are to be eliminated through the 2021 reforms, then the government must address this gap, to which the article now turns.

## 5. Transforming Perceptions: Administration Regulations 2021 and Enhanced Preview Rights

While expectation gaps have been studied in various fields, the article draws on the theory of expectation gaps developed in the audit industry.^195^ Like the Pre-pack Pool and evaluator, auditors review the veracity of statements that have been made by third parties and can give a range of opinions, from the positive, unmodified report to the negative, modified one. Similar to the battle over the scope of pre-pack oversight, there has been a battle over the core purpose of the audit role from its inception.^196^ Thus, insights into how to overcome the audit expectation gap provide a useful resource from which to develop recommendations for bridging the pre-pack regulatory expectation gap.

### A. Eliminating Expectation Gaps: Theoretical Insights

Though the audit gap has existed since the nineteenth century, it started to receive targeted attention in the 1970s.^197^ Over the decades since then, several researchers have sought to unpack its meaning and to proffer recommendations to reduce or eliminate its prevalence.^198^ We have come to understand that the expectation gap is complex and comprises further gaps.^199^ The gap between what the public expects auditors to achieve and what auditors can reasonably achieve is described by Porter as the *reasonableness gap*.^200^ Porter further describes the gap between what can be reasonably expected from the auditors and what they are perceived by the public to achieve as the *performance gap*.^201^ The performance gap signifies that the public perceives auditors as failing to meet even the legally required standards or those imposed by best practice guides. The expectation gap, being the difference between what the public reasonably or unreasonably expects of auditors and what they perceive the auditors achieve, thus comprises both the reasonableness and performance gaps. Within the performance gap are two further gaps. One is the gap between the expected standard of performance of auditors and their performance as perceived by society—the ‘deficient performance’ gap.^202^ The other is ‘deficient standards’, which refers to the gap between the duties that can reasonably be expected of auditors and the duties outlined in the law and professional guides.^203^

Ruhnke and Schmidt assert that the expectation gap is attributable to three factors.^204^ The first is the failure of the public to understand the role, duties and responsibilities of auditors as set out in the law and professional guidelines.^205^ Auditors argue that the expectation gap perpetuates because the public fails to understand the probabilistic nature of the audit and rely on *ex post* events to judge the performance of the auditors. It is these factors that create their unreasonable expectations of the audit role.^206^ Thus, for example, the public expect that where financial statements receive an unqualified opinion, it means that the auditee is financially sound.^207^ So, where the company fails shortly after receiving an unqualified opinion from its auditors, as in the case of Carillion, BHS and Patisserie Valerie, the public vilify the auditors.^208^ The second factor is the failure of the auditors themselves to understand what the laws and guidelines require of them, as well as their failure to perform to the required standards.^209^ These contribute to the deficient performance gap. Several studies have shown the discrepancies amongst auditors on the nature of their role.^210^ The third is the failure of the law and standard setters to set out clear and consistent standards, to communicate auditors’ responsibilities unambiguously and to reflect the public’s reasonable expectations of audit standards.^211^ These failures contribute to the expectation and performance gaps, as well as deficient standards and performance.

The effects of the audit expectation gap have included increased litigation, massive fines for audit firms and loss of jobs for auditors.^212^ More importantly, the gap engenders questions about the legitimacy of the audit function and fuels loss of confidence in the profession, both of which are reputationally damaging.^213^ Thus, efforts have been made to reduce the gap with suggested strategies grouped into the defensive^214^ and constructive approaches.^215^

The defensive approach advocates the reduction of the gap through the education of, and improved reassurances to, the public.^216^ Koh and Woo note calls for the expansion of the audit report to improve the education of the public.^217^ This involves the provision of a section that sets out the nature of the audit function, as well as the duties and responsibilities of auditors.^218^ In addition, Ruhnke and Schmidt advocate better training for auditors, with more oversight of misconduct, as well as the removal of ambiguities from audit standards.^219^ Collectively, these strategies would ensure that the public develop expectations that are consistent with the role, duties and responsibilities of auditors and that auditors are enjoined to execute their functions better. Their combined effect would be a reduction in the reasonableness and deficient performance gaps. While being a step in the right direction, the approach fails to address the deficit standards gap. Consequently, the defensive approach cannot, without more, effectively reduce the expectation gap.

The constructive approach deals substantively with the audit function. It adjusts the audit function to meet the reasonable expectations of the public. The strategies required include broadening the audit activities, strengthening the perceived independence of auditors and enhancing the performance of auditors.^220^ The constructive approach addresses the deficit standards gap, which would go a long way to reducing the expectation gap. Porter argues, nevertheless, that to close the gap, it is necessary to identify the aspects of the gap that one seeks to address, then to design an appropriate response.^221^ Accordingly, the most suitable response to all the facets of the expectation gap would involve a combination of both the defensive and constructive approaches.^222^

### B. Administration Regulations 2021: Enhanced Preview Rights

As was discussed in section 3, the pre-pack portends two main concerns: (i) the transparency concern; and (ii) the connected persons concern. While there have been several iterations of the pre-pack regulations since 2009, they have, until 2021, been voluntary. Furthermore, the regulations have lacked the right to stop bad deals. To that end, they did not provide the enhanced preview rights desired by leading unsecured creditor groups. As the audit experience reveals, to transform perceptions of the pre-pack, the Administrative Regulations 2021 must provide both a defensive and a constructive response to the call for enhanced preview rights. This requires the regulatory structure to address standards considered deficient by the unsecured creditors, while ensuring that the relevant stakeholders, including the practitioners, connected persons, purchasers of distressed entities and creditors, understand the system.

The Administration Regulations 2021 require mandatory oversight, which is extended not only to the pre-pack decision, but also to the deal. This approach evidences the intention to introduce a constructive response to the regulatory challenges that had characterised the self-regulated system. As section 5A demonstrates, a constructive approach responds both to substantive challenges in the previous regime and the expectations of the group with negative perceptions. However, only creditors have been given the power to stop a potentially bad deal, when approached by the administrator.^223^ The availability of this power would have stopped a pre-pack such as the Polestar Group.^224^ The situation is different, however, under the qualifying report option. Responses to the 2021 regulations demonstrate that administrators would much prefer the qualifying report option.^225^ This option retains the confidentiality that characterises the pre-pack. It also provides much more flexibility than the creditor option in the event of a negative response.^226^ Interestingly, unsecured creditors also seem to prefer to have an external expert review the deal. They keenly advocated for the extension of the Pre-pack Pool to other insolvency procedures. Thus, it can be expected that they would generally prefer the qualifying report option.^227^ The scale of preview rights given to the evaluator is therefore fundamental to the success of the reforms.

Unlike referrals to the Pre-pack Pool, which focused solely on the reasonableness of the pre-pack decision under the previous regime, the Administration Regulations 2021 require the evaluator to determine whether the decision to pre-pack and the consideration provided are reasonable.^228^ This approach would require a consideration of the deal, including, most importantly, the valuation question, which is at the heart of pre-pack disquiet. This is a constructive response that directly addresses aspects of the deficient standards gap that undermined the ability of the former regime to quell the disquiet. By expanding the oversight responsibilities of the evaluator to valuation as well as the pre-pack decision, it is expected that valuation concerns raised by critics would be mitigated.

Nonetheless, the Administration Regulations 2021 do not address the other prong of the enhanced preview rights, which is the ability to stop an unfavourable deal. Unlike the creditor approval route, the administrator may execute the deal even where the qualifying report returns a case not made.^229^ Given that connected persons may shop around for a favourable report, the regulations mean that the deal can be put through even where no evaluator would agree that it is reasonable. All that is required is that the administrator provides an explanation after the fact.^230^ This surely ought not to be. A deal that no evaluator would agree to is a potentially bad deal. It is not clear why the law permits the administrator to disregard the decision of the evaluator but not that of the creditors. This leeway simply incentivises practitioners to use the qualifying report option, which falls short of the enhanced review rights critics expect. The corollary is that the regulations could fail to transform the negative perceptions of the pre-pack. In particular, the signal that should give comfort to unsecured creditors—the possibility of stopping bad deals—is not present, which undermines the effectiveness of the constructive approach required to transform the system.

In addition, this approach fails to address one of the vexing results of sales of connected persons. The three detailed empirical reports on pre-packs in the UK have revealed the high rate of recidivism that characterises sales to connected persons.^231^ Such sales fail at nearly twice the rate of sales to persons with no previous connection to the distressed entity. More attention ought to be given to the fact that almost one-third of such sales fail within 36 months. Thus, it is argued that the administrator ought not to have the power to execute a deal where a negative report has been given. In such circumstances, the administrator should be required to approach the creditors. Such an approach would have stopped a pre-pack like that of the Polestar Group.

Some may argue that the pre-pack should be allowed to proceed because it would facilitate the conclusion of business rescues. Moreover, it would not give the unsecured creditors less than they would get in liquidation. These arguments are unconvincing when read in light of the purpose of the reforms. The purpose of the reforms was to address the concerns undergirding the negative perceptions of the pre-pack. As has been set out in detail above, achieving the purpose of the reforms requires a constructive response to the substantive issues that have been raised. It follows, therefore, that the reforms should enable enhanced preview rights, which include the ability to stop bad deals. It is submitted that in the small number of cases in which the ultimate qualifying report is negative, the administrator should be required to consult the unsecured creditors. This will give them the option to stop the deal, if considered a bad deal.

Much of the criticism of the Administration Regulations 2021 have focused on the omission of express qualifications that the evaluator must possess.^232^ Given that the evaluator is fundamental to the preferred qualifying report option, this has been decried by practitioners.^233^ Indeed, one component of the expectation gap is the performance gap. This refers to the ability of the professional to effectively execute the standards that have been set by the regulatory system. The imposition of express qualifications may thus have addressed this issue. Nonetheless, it is argued that the seemingly unregulated approach to evaluator selection permits persons with a wide range of experience to provide the qualifying report, including turnaround professionals and people who have had business experience. Further, the criticism from practitioners can underestimate the implications of the tweaks bolted on by the regulator.

The Administration Regulations 2021require the administrator to assure themself that the evaluator has the necessary experience, and perhaps qualifications, to make the statements set out in the qualifying report. More importantly, unlike the first iteration of the Bill, the Administration Regulations 2021 lay personal responsibility for the opinion provided at the door of the evaluator, who is expected to hold or acquire professional indemnity insurance.^234^ The expectation is that only persons of certain professional standards would be able to access such indemnity. Such is the fear of personal liability in the industry that the prospect of personal liability was largely responsible for the poor uptake of the recently abolished moratorium on Company Voluntary Arrangements for small companies.^235^ Similarly, it is possible that this concern for personal liability may have, in fact, been responsible for the limited statement provided by Pre-pack Pool experts in 2015. At the 2019 closed plenary with regulators and representatives of professional bodies, Pre-pack Pool members repeatedly raised concerns about their possible personal liability for opinions rendered.^236^ Thus, it is expected that the need for insurance indemnity would signal the quality and experience required to perform the role of evaluator, as well as the prospect that unsecured creditors have of holding them to account. Thus, there is a reasonable level of checks in place.

Notwithstanding the foregoing, there are additional reasons why a clearly delineated body of evaluators should be considered. While much of the foregoing discussion has focused on the constructive response to pre-pack regulation, the expectation gap insights have shown that defensive responses are also necessary to close any gap. Essentially, this means that a change in the standards must also be combined with education, as well as awareness and capacity building. Clearly, the role of the evaluator must be monitored to ensure compliance, as well as to ensure that they can be held to account by dissatisfied creditors. The challenge is that a diffuse approach has been adopted. In principle, neither evaluators nor connected persons would be repeat players. On one hand, this may reduce the likelihood of capture, but on the other hand, it makes it difficult to combine the defensive element with the constructive. Thus, it is imperative that empirical assessments of the reforms are undertaken regularly, to inform the future direction of the regulatory framework. This would be better achieved where there is an identifiable body of evaluators. At the least, evaluator reports should be reviewed at three-year intervals, instead of the focus on a single year, as has been done by the regulator through the Pre-pack Report 2020 or through empirical investigations commissioned by R3. As can be observed from the Wolverhampton Report, a 36-month review of the trajectory of rescued businesses is required to gauge the likelihood of any pre-packed business surviving. To determine the success or failure of recommendations, therefore, any review should consider data over a period of at least 36-months. That timeline would also better assist the regulator in determining the effect of the absence of the viability review.

Finally, the regulator should improve the defensive response by reiterating the importance of compliance with the important elements of disclosure documents by practitioners. This should improve the disclosures around the marketing practice. As seen, practitioners have complied more with elements showing a lack of marketing than with elements revealing attempts to market the business. Also, the regulator should incorporate dialogue-building activities with stakeholders and the public. This would include targeted communications with succinct insights into the pre-pack practice using the information that is now disclosed through various documents. The combination of constructive and defensive responses would be necessary to remedy negative perceptions of the pre-pack, as has been found in audit practice.

## 6. Conclusion

Unless they are banned, which looks unlikely, pre-packs are here to stay. However, the government appears committed to eradicating the negative perceptions plaguing the practice. As this article has shown, this can be achieved only by recognising, understanding and remedying the key concerns at the heart of these perceptions. Proponents of the pre-pack practice historically focused solely on the transparency concerns that fuel negative perceptions of the practice. Pursuant to this, they advocated only a defensive response to the concerns raised. This approach led to the introduction of regulatory technologies aimed at informing stakeholders after the conclusion of the deal and building the capacity of practitioners to execute pre-pack deals within the ethical limits advocated by the profession. These remedies proved insufficient, however, as they were ineffectual in stemming the tide of persistent criticisms of the pre-pack practice. Conversely, critics of the process focused on both the transparency and connected persons concerns. Thus, they have consistently demanded the right to *preview* the pre-pack deal and the consideration paid, as well as the power to stop a perceived bad deal. They have been clear about the comfort they would derive from reforms based on these expectations. As such, the Administration Regulations 2021 offer, for the first time, a constructive response to pre-pack concerns as they close the gaps between the expectations of the stakeholders most affected by the practice and the standards expected of practitioners. The Regulations are thus expected to go a long way to stemming the negative perception of the pre-pack.

Notwithstanding, there are additional concerns to which the regulator ought to direct attention. Importantly, the permission given to the practitioner to complete deals that have received a negative comment from the evaluator undermines the comfort that the Regulations ought to provide to unsecured creditors. The Pre-pack Pool experience showed that all but one of the companies that received a negative response failed subsequently. Thus, greater attention should be given to the link between recidivism and a negative response from the evaluator. One way to address this concern is to require the administrator to consult the creditors where they wish to sell against the opinion of the evaluator. Reducing the rate of recidivism of pre-packed entities, particularly those sold to connected persons, would reduce the negative perceptions of the practice. Tackling recidivism is thus the last frontier in improving pre-pack perceptions and it ought to receive adequate attention from the policy maker.

In addition, it is important that the regulator takes leadership of the development of the role of the evaluator, which would require a more focused group of evaluators, as well as systematic reviews of evaluators’ reports every three years to gain clearer insights into the trajectory of rescued businesses. Finally, the regulator must incorporate continuous capacity-building for evaluators and education for all stakeholders, as well as the public, if the negative perceptions of the pre-pack are to be reduced.

## Footnotes

1 B Jackson, ‘Pre-packs and SIP 16’ (2010) (Spring) Recovery 31; N Craven and L Mills, ‘“Pre-pack” Deals Risk Cheating Creditors: Private Equity Boss Warns of Abuse over “Quickie Bankruptcy”’ (*ThisIsMoney*, 5 January 2009); A Armstrong, ‘Why Pre-packs Are Still Dividing Opinion’ *The Telegraph* (London, 6 May 2017).

2 V Finch, ‘Pre-packaged Administrations: Bargains in the Shadow of Insolvency or Shadowy Bargains?’ [2006] JBL 568.

3 T Astle, ‘Pack Up Your Troubles: Addressing the Negative Image of Pre-packs’ (2015) 28 Insolvency Intelligence 72; A Bloom and S Harris, ‘Pre-packaged Administrations—What Should Be Done Given the Current Disquiet?’ (2006) 8 Insolvency Intelligence 122; M Parkhouse and K Scott, ‘A Fair Deal? (2009) <https://www.newlawjournal.co.uk/content/fair-deal> accessed 26 July 2021. For examples of media stories, see J Guthrie, ‘Debt Dodgers Revel in Return of the Phoenix’ *Financial Times* (London, 21 January 2009); L Haddou and J Cumbo, ‘Companies Use “Pre-packs” to Dump £3.8bn of Pension Liabilities’ *Financial Times* (London, 9 April 2017).

4 The Administration (Restrictions on Disposal etc to Connected Persons) Regulations 2021 (Administration Regulations 2021).

5 Insolvency Act 1986, Sch B1.

6 ibid para [2].

7 ibid paras [2], [3] and [6].

8 ibid para [49].

9 ibid para [51].

10 ibid para [52](1).

11 Finch (n 2).

12 Statement of Insolvency Practice 16 (SIP 16) <https://insolvency-practitioners.org.uk/uploads/documents/f30389ce35ed923c06b2879fecdb616a.pdf> accessed 9 November 2021.

13 Insolvency Act 1986, Sch B1, para [2]; S Frisby, ‘A Preliminary Analysis of Pre-packaged Administrations’ Report to R3—The Association of Business Recovery Professionals (August 2017, R3 – The Association of Business Recovery Professionals) 18.

14
*Re Christophorus 3 Limited* [2014] EWHC 1162 (Ch).

15
*Re Kayley Vending Ltd* [2009] EWHC 904.

16
*Re Nationwide Accident Repair Services* [2020] EWCH 2420 (Ch).

17
*Kayley Vending* (n 15); Frisby, ‘Preliminary Analysis’ (n 13) 19–20.

18 Enterprise Act 2002. See P Walton, ‘Pre-packaged Administrations—Trick or Treat?’ (2006) 19 Insolvency Intelligence 113.

19 Frisby, ‘Preliminary Analysis’ (n 13) 5; Bloom and Harris (n 3) 122–3.

20 T Graham, ‘Graham Review into Pre-pack Administration: Report to the Rt Hon Vince Cable MP’ (June 2014) 4–5 (Graham Review) <www.gov.uk/government/publications/graham-review-into-pre-pack-administration> accessed 13 July 2021.

21 J Moulton, ‘The Uncomfortable Edge of Propriety-Pre-packs or Just Stitchups?’ (2005) (Autumn) Recovery 2; but see S Frisby, ‘Insolvency Law and Insolvency Practice: Principles and Pragmatism Diverge?’ (2011) 64 CLR 349, 396.

22 See Section 4.A below.

23 Frisby, ‘Preliminary Analysis’ (n 13) 28.

24 ibid 4.

25 Graham Review (n 20).

26 Prepack Sales in Administration Report 2020 (Pre-pack Report 2020) s 7.3. <www.gov.uk/government/publications/pre-pack-sales-in-administration/pre-pack-sales-in-administration-report> accessed 26 July 2021.

27 SBEE Act, s 129, which inserted the Insolvency Act 1986, Sch B1, para [60A][10]. The power was extended to June 2021 by the Corporate Insolvency and Governance Act 2020, s 8 of which introduced a new Insolvency Act 1986, Sch B1, para [60A][10].

28 Pre-pack Report 2020 (n 26) s 7.

29 Ibid. See 6 below.

30
*DKLL Solicitors v HMRC* [2008] 1 BCLC 112; P Walton, ‘Pre-packin’ in the UK’ (2009) 18 International Insolvency Review 85.

31 Frisby, ‘Preliminary Analysis’ (n 13) 28.

32 S Frisby, ‘Pre-packaged Administrations: Progress Report to the Association of Business Recovery Professionals’ (2008) 30.

33 P Walton, C Umfreville and P Wilson, ‘Pre-pack Empirical Research: Characteristic and Outcome Analysis of Pre-pack Administration’ (2014) (Wolverhampton Report) <www.gov.uk/government/publications/graham-review-into-pre-pack-administration> accessed 13 July 2021.

34 ibid 33.

35 ibid 33.

36 ibid 66.

37 Graham Review (n 20) 49.

38 V Finch, ‘Pre-packaged Administration and Construction of Propriety’ (2011) 11 JCLS 1.

39 See Section 4.B below.

40 Graham Review (n 20) s 8.

41 ibid 48.

42 ibid 48.

43 ibid 66.

44 Pre-pack Report 2020 (n 26) 14–15.

45 Where 62% bought at or over the valuation figure but 7% exactly matched it, only 55% paid above. Pre-pack Report (n 26) 15.

46 ibid 15.

47 ibid 15.

48 Graham Review (n 20) 45–7.

49 Pre-pack Report 2020 (n 26) 13.

50 ibid 13.

51 ibid 13–14. For the principles, see Graham Review (n 20) 64–6.

52 Pre-pack Report 2020 (n 26) 13–14.

53 ibid 14.

54 ibid 14.

55 ibid 14.

56 Graham Review (n 20) 51.

57 ibid 62–3.

58 ibid 59–62.

59 ibid 62–3.

60 SIP 16 (n 12).

61 Pre-pack Report 2020 (n 26) 12.

62 ibid 12.

63 Wolverhampton Report (n 33) 36.

64 ibid 35.

65 Pre-pack Report 2020 (n 26) 12.

66 Graham Review (n 20) 59.

67 Pre-pack Pool, ‘About the Pool’ <www.prepackpool.co.uk/about-the-pool> accessed 16 July 2021. On the role of the Pool, see B Adebola, ‘Proposed Feasibility Oversight for Pre-pack Administration in England and Wales: Window Dressing or Effective Reform? (2015) 8 JBL 591.

68 Graham Review (n 20) 37.

69 Pre-pack Report (n 26) 10.

70 ibid 10.

71 ibid 11.

72 ibid 11.

73 ibid 10. See also: B Adebola, ‘The Case for Mandatory Referrals to the Pre-pack Pool’ (2019) 32(2) Insolvency Intelligence 71.

74 Pre-pack Report 2020 (n 26) 10.

75 ibid 19.

76 See <https://insolvency-practitioners.org.uk/regulation-and-guidance/england-wales/> accessed 16 July 2021.

77 Pre-pack Report 2020 (n 26) 17.

78 The proponents are set out in the following paragraph.

79 Bloom and Harris (n 3).

80 Insolvency Service, ‘Improving the Transparency of, and Confidence in, Pre-packaged Sales in Administration: Summary of Consultation Responses’ (2011) (Improving Pre-packs Consultation) para 2.2.6.

81 Bloom and Harris (n 3) 122–3.

82 ibid 123; Improving Pre-packs Consultation (n 80) 29.

83 Improving Pre-packs Consultation (n 80) 24.

84 See eg ‘Comments by the Association of Business Recovery Professionals (“R3”) in Response to the Consultation Document Issued by the Insolvency Service in March 2010’ <https://www.r3.org.uk/stream.asp?stream=true&eid=22197&node=198&checksum=696C372EA201BE183127E8560EA5B8F7> accessed 26 July 2021; evidence of ICAEW in Business, Innovation and Skills Committee, *The Insolvency Service* (HC 2012–13, 675) 22, Ev 69.

85 Pre-pack Report 2020 (n 26), Executive Summary.

86 Improving Pre-packs Consultation (n 80) 39.

87 ibid 39; HC 675 (n 84) 22.

88 ibid 7–11.

89 ibid 21.

90 Bloom and Harris (n 3) 122–3.

91 Improving Pre-packs Consultation (n 80) 29–30.

92 ibid 29–30.

93 ibid 36.

94 ibid 36.

95 ibid 33.

96 ibid 38.

97 Other vocal creditor groups include the Institute of Credit Management and the Road Haulage Association.

98 House of Commons Business and Enterprise Committee, *The Insolvency Service* (HC 2008-09, 198) 12.

99 Improving Pre-packs Consultation (n 80) 38. For similar opinions from the BPIF, House of Commons Business, Innovation and Skills Committee, ‘The Insolvency Service’ (2013) HC 675, Ev 34.

100 Emphasis added. HC 198 (n 98) 14.

101 SIP 16 (2009) <https://insolvency-practitioners.org.uk/uploads/documents/5e680cfcf2e5c095e1057b526e8cf953.pdf> accessed 10 November 2021. SIP 13 was introduced in 2016 <https://insolvency-practitioners.org.uk/uploads/documents/7ac06c8bd637a1c82f64916ba0f4a8f0.pdf> accessed 10 November 2021.

102 ibid.

103 On the Joint Insolvency Committee, see <https://insolvency-practitioners.org.uk/regulation-and-guidance/joint-insolvency-committee-jic/> accessed 10 November 2021. On the Association of Business Recovery Professionals, see <www.r3.org.uk/> accessed 10 November 2021. For recognised professional bodies, see <www.gov.uk/government/publications/insolvency-practitioners-recognised-professional-bodies/recognised-professional-bodies> accessed 10 November 2021.

104 See section 3 above.

105 For examples of reports, see <www.gov.uk/government/publications/statements-of-insolvency-practice-16-sip-16> accessed 10 November 2021.

106 See above (n 101) 1.

107 We are currently on the fourth iteration of the SIP 16 <www.icaew.com/regulation/insolvency/sips-regulations-and-guidance/statements-of-insolvency-practice/statements-of-insolvency-practice-sips-england> accessed 26 July 2021.

108 For latest version, see <https://insolvency-practitioners.org.uk/regulation-and-guidance/england-wales/> accessed 24 May 2022.

109 Graham Review (n 20) 4.

110 Read ibid in conjunction with the Wolverhampton Report (n 33).

111 Graham Review (n 20) 66.

112 ibid 62.

113 ibid 64.

114 See the 2015 iteration of SIP 16: <www.r3.org.uk/technical-library/england-wales/sips/more/29131/page/1/sip-16-pre-packaged-sales-in-administrations/> accessed 26 July 2021.

115 Graham Review (n 20) 59.

116 See above (n 114).

117 See Pre-pack Pool (n 67).

118 See 9 above.

119 See Questions & Answers about the Pre-pack Pool <www.prepackpool.co.uk/questions-answers> accessed 3 April 2020.

120 The previous Insolvency Code of Ethics was effective 1 January 2009–30 April 2020. The New Code is effective from 1 May 2020.

121 T Stanhope, ‘Ethics Code for Insolvency Practitioners’ <www.guildhallchambers.co.uk/files/EthicsCodeInsolvencyPractitioners_ChrisBrockman&TracyStanhope.pdf> accessed 26 July 2021.

122
*Sisu Capital Fund Limited v Tucker* [2006] BPIR 154.

123 Insolvency Code of Ethics, para 51.

124 2370.3 A2, ICAEW, Insolvency Code of Ethics <www.icaew.com/-/media/corporate/files/technical/ethics/insolvency-code-of-ethics.ashx?la=en > accessed 26 July 2021.

125 Insolvency Code of Ethics, para 39.

126 For an earlier prediction of the ineffectiveness of the Pool, see Adebola, ‘Proposed Feasibility’ (n 67).

127 Questions & Answers about the Pre-pack Pool (n 119).

128 ibid, Question 23.

129 Pre-Pack Pool, ‘Pre-pack Pool—Annual Report 2018’ 3 <www.prepackpool.co.uk/uploads/files/documents/Pre-Pack-Pool-2018-Annual-report-v4.pdf> accessed 26 July 2021.

130 ibid 4.

131 ibid 4.

132 Frisby, ‘Preliminary Analysis’ (n 13) 43.

133 On the changes, see I Fletcher, ‘UK Corporate Rescue: Recent Developments—Changes to Administrative Receivership, Administration, and Company Voluntary Arrangements—the Insolvency Act 2000, the White Paper 2001, and the Enterprise Act 2002’ (2004) 5 EBOR 119.

134 ibid 45.

135 Wolverhampton Report (n 33) 19.

136 Pre-pack Report 2020 (n 26) 10.

137 The same arguments can be made for the pre-pack sale to unconnected persons.

138 Frisby, ‘Preliminary Analysis’ (n 13) 26; Wolverhampton Report (n 33) 21 and 84.

139 Frisby, ‘Preliminary Analysis’ (n 13) 31.

140 Wolverhampton (n 33) 23 and 84.

141 ibid 84.

142 S Frisby, ‘Report on Insolvency Outcomes: Presented to the Insolvency Service’ (June 2006) 11.

143 L Conway, ‘Regulation of Insolvency Practitioners (IPs)’ (2019) 6 <https://commonslibrary.parliament.uk/research-briefings/sn05531/> accessed 3 April 2020.

144
*Dunbar Assets Plc v Davey* [2018] EWHC 766 (CH); *Brewer and Another v Iqbal* [2019] EWHC 182 (CH)

145 On agency capture, see: D. Carpenter and D. Moss (eds)*, Preventing Regulatory Capture: Special Interest Influence and How to Limit It* (CUP 2014).

146 Frisby, ‘Report on Insolvency Outcomes’ (n 143) 11.

147 Frisby, ‘Preliminary Analysis’ (n 13) 6.

148 J Armour, A Hsu and A Walters, ‘The Costs and Benefits of Secured Creditor Control in Bankruptcy: Evidence from the UK’ (2006) 7 (Working Papers wp332, Centre for Business Research, University of Cambridge) accessed 3 November 2021. See also Frisby, ‘Report on Insolvency Outcomes’ (n 143).

149 S Frisby, ‘Pre-packaged Administrations: Progress Report to the Association of Business Recovery Professionals’ (2008) 26.

150 ibid 27.

151 ibid 27.

152 See B Adebola, ‘An Invitation to Encourage Due Consideration for the Survivability of Rescued Businesses in the Business Rescue System of England and Wales’ (2017) 26 International Insolvency Review 129. See also *Kayley Vending* (n 15).

153 On what happens when administrator’s proposals are rejected, see B Adebola, ‘Discretion or Obligation to Seek Directions: The Administrator and Rejected Proposals’ (2014) 1 NIBLeJ 2.

154 ‘A Fair Deal? Mark Parkhouse & Kerry Scott on the Criticism of Pre-package Administrations’ <https://www.newlawjournal.co.uk/content/fair-deal>.

155 For a strongly worded argument for quality of the price paid by previously connected persons, see ‘The Graham Pre-pack Review—Why Is Everyone Cheering?’ <https://complianceoncall.blogspot.com/2014/07/the-graham-pre-pack-review-why-is.html> accessed 26 July 2021.

156 Graham Review (n 20) 48. See also 6 above.

157 For the list of documents to submit, see Guidance Documents <www.prepackpool.co.uk/guidance-documents> accessed 26 July 2021.

158 Accountancy Age, ‘The Pre Pack Pool: Problem-Prone, Time-Consuming, Costly and Pointless’ <https://www.accountancyage.com/2016/04/08/the-pre-pack-pool-problem-prone-time-consuming-costly-and-pointless/> accessed 26 July 2021.

159 S Moppett, ‘An IP’s Perspective of the Pre Pack Pool’ <https://krecr.co.uk/an-ips-perspective-of-the-pre-pack-pool/> accessed 26 July 2021.

160 On pre-pack pool process, see Questions & Answers about the Pre-pack Pool (n 119); Graham Review (n 20) 62.

161 Pre-pack Pool (n 67) 4.

162 G Ruddick, ‘UK’s Largest Independent Printer Polestar Calls in Administrators’ *The Guardian* (London, 27 April 2016).

163 S Mundy, ‘Pension Protection Fund faces Polestar Test’ *Financial Times* (London, 28 Octoboer 2011); between 1998 and 2011, Polestar had reportedly undergone four pre-packs: S Munday, ‘Retailers Eye Controversial Pre-pack Deals’ *Financial Times* (London, 14 September 2011).

164 G Ruddick, ‘About 2,000 Jobs at Risk as Printer Polestar Battles Funding Crisis’ *The Guardian* (London, 4 December 2015); J Francis, ‘Polestar Sites Go into Administration’ (*PrintWeek*, 25 April 2016); ‘Proventus Takes Firm Grip After Polestar Administration’ (*Print Business*, 4 April 2016).

165 PWC, ‘Joint Administrators’ Proposals for Achieving the Purpose of Administration’ (2016) 6 <www.pwc.co.uk/business-recovery/administrations/assets/polestar-proposals.pdf> accessed 26 July 2021.

166 ibid 6.

167 L Kilganon, ‘Shortfall to Top £300m at Magazine Printer’ <www.insidermedia.com/insider/national/shortfall-to-top-300m-at-magazine-printer> accessed 26 July 2021.

168 N Mansley, ‘2016 and All That … Polestar: Six Months of Mayhem (*PrintWeek*, 12 December 2016).

169 E Vaccario, ‘Pre-Pack Pool: Which Way Forward?’ Presentation Made to the Reviewers’ Meeting, Pre-pack Pool Ltd (2019). Copy on file with author.

170 ‘Proventus Steps in to Buy “Sound” Polestar Business’ (*Print Business*, 13 December 2015).

171 See 12 above.

172 Anecdotal evidence suggests that it was known even to the Pool that the company was unlikely to survive.

173 J Braithwaite, *To Punish or Persuade: Enforcement of Coal Mine Safety* (State University of New York Press 1985) 124–5.

174 I Ayres and J Braithwaite, *Responsive Regulation* (OUP 1992) 38–9. On regulation, see R Baldwin, M Cave and M Lodge, *The Oxford Handbook of Regulation* (OUP 2010).

175 Administration Regulations 2021, r 1.

176 Ibid r 3(1) and 3(2).

177 Insolvency Service, ‘Guidance: Requirements for Independent Scrutiny of the Disposal of Assets in Administration, including Pre-pack Sales’ (Insolvency Service Guidance 2021) (30 April 2021) <www.gov.uk/government/publications/requirements-for-independent-scrutiny-of-the-disposal-of-assets-in-administration-including-pre-pack-sales/requirements-for-independent-scrutiny-of-the-disposal-of-assets-in-administration-including-pre-pack-sales#substantial-disposal> accessed 20 July 2021.

178 Administration Regulations 2021, r 4(2)(a).

179 ibid r 4(2)(b).

180 Insolvency Service Guidance 2021, s 7.

181 Administration Regulations 2021, r 7(b).

182 ibid r 7(c) and 11; Insolvency Service Guidance 2021, s 8.

183 Administration Regulations 2021, r 13.

184 Insolvency Service Guidance 2021, s 7.

185 Administration Regulations 2021, r 6 and 7; Insolvency Service Guidance 2021, ss 6 and 8.

186 Administration Regulations 2021, r 7(h)(i); Insolvency Service Guidance 2021, s 8.

187 Administration Regulations 2021, r 7(h)(ii); Insolvency Service Guidance 2021, s 8.

188 Administration Regulations 2021, r7(i).

189 Insolvency Service Guidance 2021, s 9.

190 ibid s 7.1.

191 ibid s 9.

192 Insolvency Act 1986, Sch B1, para [4].

193 Administration Regulations 2021, r 9(3) and 9(4); Insolvency Service Guidance 2021, s 9.

194 See 12 above.

195 In political science, R Waterman, C Silva, and H. Jenkins-Smith, *The Presidential Expectations Gap: Public Attitudes Concerning the Presidency* (University of Michigan Press 2014); in public sector management, M Meyer-Höfer, S Nitzko and A Spiller, ‘Is There an Expectation Gap? Consumers’ Expectations towards Organic: An Exploratory Survey in Mature and Emerging European Organic Food Markets’ (2015) 117 British Food Journal 1527.

196 R Chandler and others, ‘Changing Perceptions of the Role of the Company Auditor, 1840–1940’ (1993) 23 Accounting and Business Research 443, 444.

197 C Liggio, ‘The Expectation Gap: The Accountant’s Legal Waterloo?’ (1974) 3 Journal of Contemporary Business, 27.

198 For a brief overview, see R Quick, ‘The Audit Expectation Gap: A Review of Academic Literature’ (2020) 94 Maandblad Voor Accountancy en Bedrijfesrconomie 5.

199 Other gaps that have been identified. For example, the materiality gap in R Boterenbrood, ‘The Audit Expectation Gap between Companies and Their Auditors: An Exploratory Study’ (2017) 18 Global Business Review 1124, 1130; the knowledge gap and the evolution gap in A Diolas, ‘Closing the Expectation Gap in Audit’ <www.accaglobal.com/in/en/professional-insights/global-profession/expectation-gap.html> accessed 10 April 2020.

200 B Porter, ‘An Empirical Study of the Audit Expectation–Performance Gap’ (1993) 24 Accounting and Business Research 49, 50.

201 ibid 50.

202 ibid 50.

203 ibid 50.

204 K Ruhnke and M Schmidt, ‘The Audit Expectation Gap: Existence, Causes, and the Impact of Changes (2014) 44 Accounting and Business Research 572, 575.

205 ibid 575.

206 C Humphrey, P Moizer and S Turley, ‘The Audit Expectations Gap in Britain: An Empirical Investigation’ (1993) 23 Accounting and Business Research 395.

207 See, however, FRC, ‘International Standard on Auditing (UK and Ireland) 200’ (revised June 2016, updated January 2020) A1, para [3].

208 J Jolly, ‘“Decline in Quality”: Auditors Face Scrutiny over String of Scandals’ *The Guardian* (London, 1 February 2019).

209 Ruhnke and Schmidt (n 207) 575.

210 See eg Porter (n 203) 51–3.

211 Ruhnke and Schmidt (n 207) 575.

212 S White, ‘Regulator Hands Out Record £43m Fines for Bad Audit’ (*Accountancy Daily* 31 July 2019) <www.accountancydaily.co/regulator-hands-out-record-ps43m-fines-bad-audit> accessed 11 April 2020; but see E Cowle, S Rowe, ‘Don’t Make Me Look Bad: How the Audit Market Penalizes Auditors for Doing Their Job’ (2018) <https://papers.ssrn.com/sol3/papers.cfm?abstract_id=3228321> accessed 11 April 2020.

213 M Vincent, ‘KPMG Pays £10m in Fines but Auditors Owe the Public More’ *Financial Times* (London, 8 May 2019); Runhke and Schmidt (n 207) 573.

214 Alternatively, the ‘protective’ approach. Humphrey, Moizer and Turley (n 209) 145.

215 ibid 145.

216 ibid 145. See also B Pierce and M Kilcommins, ‘The Audit Expectations Gap: The Role of Auditing Education’ (1993) <https://core.ac.uk/download/pdf/11308641.pdf> accessed 10 April 2020.

217 H Koh and E Woo, ‘The Expectation Gap in Auditing’ (1998) 13 Managerial Auditing Journal 147, 150–1.

218 In the UK, see FRC, ‘Extended Auditor’s Reports: A Review of Experience in the First Year’ (2015) Appendix 1 <www.frc.org.uk/getattachment/561627cc-facb-431b-beda-ead81948604e/Extended-Auditor-Reports-March-2015.pdf> accessed 12 April 2020.

219 Ruhnke and Schmidt (n 207) 575.

220 Humphrey, Moizer and Turley (n 209) 152; Quick (n 201) 7.

221 Porter (n 203) 66.

222 Ruhnke and Schmidt (n 207) 592–4.

223 See 24 above.

224 See 22 above.

225 eg ‘Second Time Lucky: Pre-pack Reform in the UK’ (October 2020) <www.engage.hoganlovells.com/knowledgeservices/attachment_dw.action?attkey=FRbANEucS95NMLRN47z%2BeeOgEFCt8EGQJsWJiCH2WAWBW0hl3oTMYqHo63bs5qmG&nav=FRbANEucS95NMLRN47z%2BeeOgEFCt8EGQbuwypnpZjc4%3D&attdocparam=pB7HEsg%2FZ312Bk8OIuOIH1c%2BY4beLEAeRskAq1cWE2I%3D&fromContentView=1> accessed 26 October 2021.

226 See 25 above.

227 Pre-pack Report 2020 (n 26) 11.

228 Administration Regulations 2021, r 7(h)(i); Insolvency Service Guidance 2021, s 8.

229 See 25 above. See also Insolvency Service Guidance 2021, s 8.

230 See section 2A above.

231 See sections 2 and 3 above.

232 ‘Draft Pre-pack Rules Set Out Role of Evaluator’ (*Accountancy Daily*, 25 February 2021) <www.accountancydaily.co/draft-pre-pack-rules-set-out-role-evaluator> accessed 26 July 2021.

233 ibid.

234 Administration Regulations 2021, rr 7(c) and 11; Insolvency Service Guidance 2021, s 8.

235 Insolvency Service, ‘Insolvency and Corporate Governance: Government Response’ (26 August 2018) 45 <https://www.gov.uk/government/consultations/insolvency-and-corporate-governance> accessed 26 July 2021.

236 This author was present at the meeting.

## Notes

Many thanks to the anonymous reviewers for their comments. Special thanks to the attendees of the Insolvency Service Forward Thinking Conference, as well as Dr Jorge Guira, Prof Chris Hilson and Dr Titilayo Adebola for their comments on earlier drafts of the article.

